# Biofilm Formation of Clinical *Klebsiella pneumoniae* Strains Isolated from Tracheostomy Tubes and Their Association with Antimicrobial Resistance, Virulence and Genetic Diversity

**DOI:** 10.3390/pathogens10101345

**Published:** 2021-10-18

**Authors:** Dorota Ochońska, Łukasz Ścibik, Monika Brzychczy-Włoch

**Affiliations:** 1 Department of Molecular Medical Microbiology, Chair of Microbiology, Faculty of Medicine, Jagiellonian University Medical College, 18 Czysta Street, 31-121 Krakow, Poland; 2 Department of Otolaryngology and Oncological Surgery of the Head and Neck, 5th Military Hospital with Polyclinic in Krakow, 1-3 Wrocławska Street, 30-901 Krakow, Poland; l.scibik@5wszk.com.pl

**Keywords:** biofilm, *Klebsiella pneumoniae*, PFGE, PCR, SEM, tracheostomy tube

## Abstract

(1) Background: Due to the commonness of tracheotomy procedures and the wide use of biomaterials in the form of tracheostomy tubes (TTs), the problem of biomaterial-associated infections (BAIs) is growing. Bacterial colonization of TTs results in the development of biofilms on the surface of biomaterials, which may contribute to the development of invasive infections in tracheostomized patients. (2) Methods: Clinical strains of *K. pneumoniae*, isolated from TTs, were characterized according to their ability to form biofilms, as well as their resistance to antibiotics, whether they harbored ESβL genes, the presence of selected virulence factors and genetic diversity. (3) Results: From 53 patients, *K. pneumoniae* were detected in 18 of the TTs examined, which constituted 34% of all analyzed biomaterials. Three of the strains (11%) were ESβL producers and all had genes encoding CTX-M-1, SHV and TEM enzymes. 44.4% of isolates were biofilm formers, SEM demonstrating that *K. pneumoniae* formed differential biofilms on the surface of polyethylene (PE) and polyvinyl chloride (PVC) TTs in vitro. A large range of variation in the share of fimbrial genes was observed. PFGE revealed sixteen genetically distinct profiles. (4) Conclusions: Proven susceptibility of TT biomaterials to colonization by *K. pneumoniae* means that the attention of research groups should be focused on achieving a better understanding of the bacterial pathogens that form biofilms on the surfaces of TTs. In addition, research efforts should be directed at the development of new biomaterials or the modification of existing materials, in order to prevent bacterial adhesion to their surfaces.

## 1. Introduction

Tracheotomy is one of the oldest and most often conducted otolaryngological surgical procedures. It involves the creation of an opening in the front wall of the trachea so that a tracheostomy tube can be inserted into the lumen of the airways [1,2,3]. Healthcare-associated infections (HCAIs) developing in patients with tracheostomy are a significant problem for modern medicine and are much more common in this group of patients than in others. The risk of acquiring an infection in these individuals is related to the presence of predisposing factors, as well as the increased invasiveness of diagnostic and therapeutic procedures, staff behavior and the growing resistance of microorganisms to commonly used antibiotics [4]. It should be emphasized that patients with tracheostomies are colonized by a variety of bacterial microflora as a result of massive colonization of the tracheostomy tube. Along with insufficient care of the patient, this may lead to a relocation of the microorganisms in the lower parts of the respiratory tract and thence to the initiation of the inflammatory process [1,5]. The most common infections reported among patients with tracheostomies are respiratory tract infections manifesting as ventilator-associated pneumonia (VAP), which can be expected to develop in 4–28% of patients [1,4,6,7].

Other important complications following this procedure include wound infections around the stomata and the possibility of the development of chronic otitis media with effusion, which is commonly observed in Ear, Nose and Throat (ENT) units [1,2,8,9]. A clinically significant group of infections are those closely related to the biomaterials applied in modern medicine (biomaterial-associated infections—BAIs). The ability of bacteria to form biofilm plays a key role in the pathogenesis of infections associated with the use of biomaterials, which form a susceptible surface for microbial colonization [10]. It is estimated that approximately 60–70% of hospital-acquired infections stem from the formation of biofilms on the surface of biomaterials used [11]. Diagnosing infections associated with biofilm formation on the surface of biomaterials is often problematic, as well as time-consuming [4]. Compared to free-flowing cells, microorganisms growing in a biofilm are much less sensitive to the action of antibiotics, antiseptics and the defense mechanisms of the human body [12]. Therefore, biofilm-associated infections can lead to numerous therapeutic complications which often become chronic, and which may recur after recovery [12]. Symptoms of chronic infections may be non-specific and reveal themselves only after a considerable amount of time. Chronic infections may eventually require removal and replacement of implanted devices or products, including tracheostomy tubes [13].

Bacterial biofilms are present in more than 90% of tracheostomy tubes collected in both inpatient and outpatient settings [12]. All indwelling tracheostomy tubes are contaminated with normal and sometimes pathogenic flora [1,8]. A wide spectrum of pathogenic microorganisms have the ability to adhere, colonize and, consequently, to form biofilms on the surface of tracheostomy tubes [14,15]. According to current data available in the literature, *K. pneumoniae* remains one of the most frequently reported bacterial pathogens out of all Gram-negative microorganisms capable of forming biofilms on these biomaterials, and therefore, they are the predominant bacilli isolated from tracheostomized patients exhibiting colonization, and the predominant etiological factors in the pathogenesis of infections in hospitalized patients undergoing tracheostomy procedures [4,16,17,18].

*K. pneumoniae* is an opportunistic pathogen responsible for hospital-acquired and community-acquired infections, such as pneumonia, urinary tract infection (UTI) and pyogenic liver abscess (PLA) [10]. The bacterium is able to form biofilms, i.e., aggregates of bacterial cells which embed within a self-produced matrix consisting of an extracellular polymeric substance (EPS) and which adhere to each other and/or to a surface. EPS is a complex structure comprising polysaccharides, proteins and DNA [12]. Important virulence factors that contribute to biofilm formation in *K. pneumoniae* are the capsular polysaccharides and type 1 and type 3 fimbriae [10]. Type 3 fimbriae are mainly composed of subunits of the protein MrkA and instigate biofilm formation [10]. Another protein, MrkD, which is found at the tip of fimbriae, gives the appendages their adhesive properties, allowing them to bind to polyethylene (PE) and polyvinyl chloride (PVC) surfaces, and defines the fimbrial binding capacity [10]. The present study was conceived in response to the commonness of tracheostomy procedures and the sparse data on the characteristics of the main bacterial pathogens, including the clinical strains of *K. pneumoniae* isolated from tracheostomy tubes from patients hospitalized in southern Poland. Our aim was to characterize clinical strains of *K. pneumoniae* isolated from patients with tracheostomies according to their ability to form biofilms, associated with their resistance to antibiotics, the harboring of genes for producing ESβL, the presence of selected virulence factors and genetic diversity. In addition, we assessed the formation of *K. pneumoniae* biofilms on tracheostomy tubes by determining their structure using scanning electron microscopy (SEM).

## 2. Results

### 2.1. Patients’ Clinical Characteristics

Patients’ clinical characteristics were reviewed and recorded (Table 1). During the 30 months, 18 *K. pneumoniae* isolates were collected from eighteen patients aged 77 years on average. The average duration of hospitalization was 22.4 days. Tracheostomy was performed on all patients at the Department of Otolaryngology Head and Neck Surgery (ORL) to treat laryngeal cancer, whereas, in the Department of Anesthesiology and Intensive Therapy (OAIT), it was conducted on account of the need for prolonged mechanical ventilation of the lungs. All six OAIT patients experienced VAP episodes, three of them correlated with *K. pneumoniae* pneumonia, of which one *K pneumoniae* isolate showed moderate biofilm production; the other two isolates did not produce biofilm. The remaining patients were diagnosed with pneumonia caused by *Pseudomonas aeruginosa*, *Proteus mirabilis* and *Acinetobacter baumanii*, *Staphylococcus aureus* and *P. aeruginosa* VAP recurrence occurred in one patient. VAP episodes did not occur in patients of the ORL Department because they were not undergoing ventilator therapy. Two patients died.

### 2.2. Antimicrobial Resistance Pattern

Among the *K. pneumoniae* isolates studied, 33.3% (n = 6) were resistant to cefuroxime; 27.8% (n = 5) to trimethoprim–sulfamethoxazole; 22.2% (n = 4) to ciprofloxacin, levofloxacin, moxifloxacin, ofloxacin, ceftriaxone and piperacillin; 16.7% (n = 3) to tobramycin, ceftazidime, cefotaxime, aztreonam and cefepime; 11% (n = 2) to netilmicin; and 5.5% (n = 1) to gentamicin. The results are presented in Table 2. *K. pneumoniae* isolates resistant to amikacin, doripenem, ertapenem, imipenem, meropenem and colistin were not found.

### 2.3. ESβL Gene Detection

**Figure 1:**
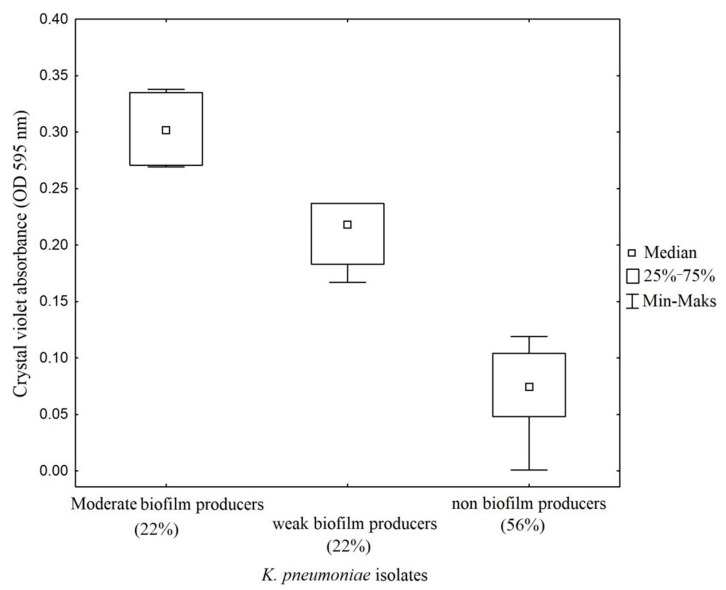
Quantitative assays of biofilm formation on abiotic surface (polystyrene microtiter plate) of clinical strains of *K. pneumoniae* isolated from patients with tracheostomies. Absorbance values of 0.254–0.509 of OD_595nm_ were classified as moderate biofilm producers. Absorbance values of 0.127–0.254 were considered as weak biofilm producers and values <0.127 were classed as non-biofilm producers.

Three (16.7%) ceftazidime and cefotaxime resistant isolates were positive for ESβL production. PCR amplification of the extended spectrum β-lactamase genes revealed that all three isolates harbored *bla*_S__HV_ and *bla*_TEM_ genes. In addition, all three isolates were positive for *bla*_CTX-M group 1_. Moreover, one of the studied isolates with a *bla*_CTX-M group 1_ also had a group 9 CTX-M (*bla*_CTX-M group 9_) ESβL (Figure 1).

### 2.4. PCR Detection of Fimbrial Genes

The PCR-based survey of *ecp*A, *fim*A, *fim*H, *mrk*A and *mrk*D fimbrial genes in the tested *K. pneumoniae* showed that 100% (n = 18) of strains carried the *mrk*A adhesin gene, while 27.8% (n = 5) carried the *fim*A gene. The *mrk*D adhesin gene was present in 38.9% (n = 7) of isolates. Moreover, 50% (n = 9) and 27.8% (n = 5) of isolates amplified *ecp*A and *fim*H genes, respectively.

### 2.5. Biofilm Production

**Figure 2:**
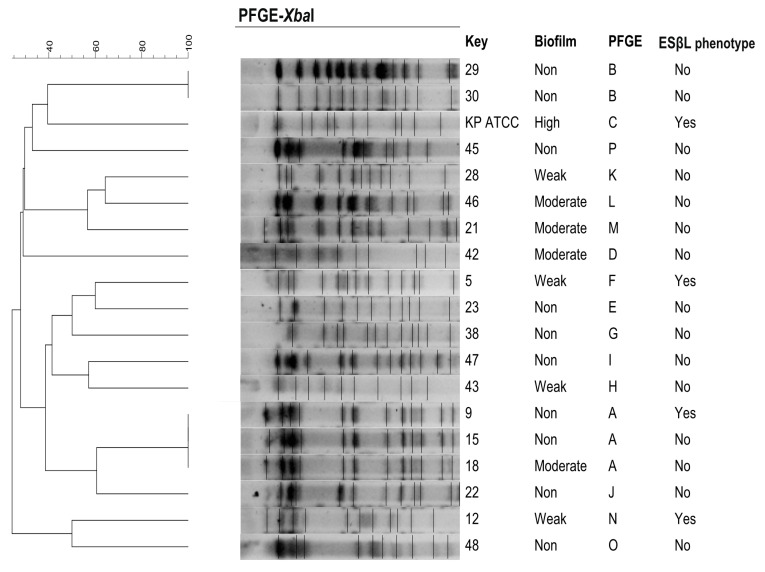
Dendrogram representing the genetic relationship between 18 *K. pneumoniae* isolates after restriction with *Xba*I enzyme. ESβL-producing strains were part of three different PFGE profiles. DNA fragments were separated by PFGE using a CHEF DR^®^II system. PFGE profiles were compared according to their percentage of similarity, estimated by the Dice coefficient and clustered by UPGMA using GelCompar II v.6.5 software. PFGE settings: optimization, 1%; tolerance, 1%. Images were captured and converted to TIFF for computer analysis.

The classification of biofilm-producing strains was established based on absorbance readings obtained from the ratio of the blank (0.069) and the positive control (1.74). Strains with absorbance readings <0.127 were considered non-biofilm formers. Absorbance values between two and four times the reading of the blank (0.127–0.254) were considered low biofilm-forming strains. Values between four and six times the blank reading (0.254–0.509) were considered medium biofilm-forming strains. When applying qualitative methods to interpret the ability of *K. pneumoniae* to form biofilm in vitro, 44.4% (n = 8) of the examined isolates were interpreted as biofilm producers and 56% (n = 10) as non-biofilm producers. Among biofilm producers, 22% (n = 4) of isolates classified as moderate biofilm producers and 22% (n = 4) of isolates identified as weak biofilm producers. Strong biofilm producers were not found (Figure 1 and Figure 2).

### 2.6. PFGE

On the basis of the chromosomal DNA patterns obtained, 16 PFGE profiles were identified among the 18 isolates. The PFGE clonal groups were designated by the letters A–P according to differences in banding patterns. The dominant PFGE clonal group types: A (16.7%, n = 3) and B (11%, n = 2) were found. The remaining isolates were clustered into 13 singletons (C–P). ESβL-producing *K. pneumoniae* were part of three different PFGE profiles (A, F and N) (Figure 2).

### 2.7. SEM

**Figure 3:**
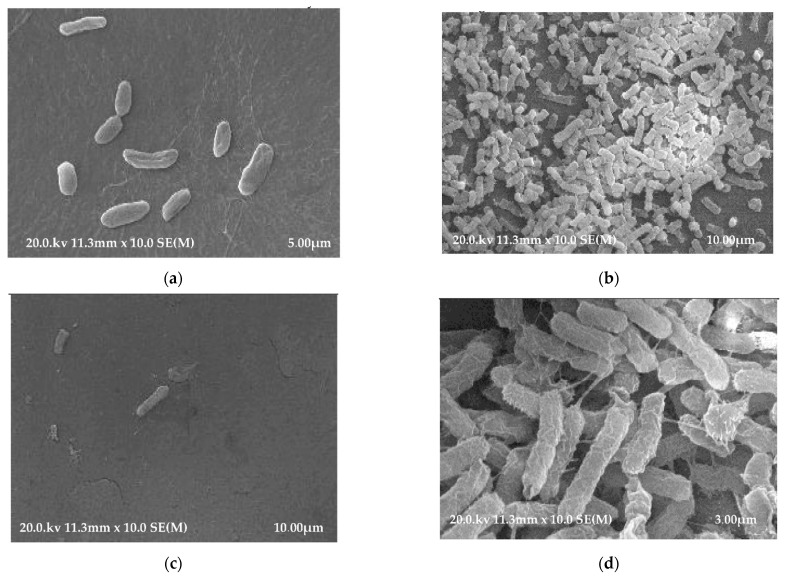
(**a**) SEM micrograph of a polyethylene tracheostomy tube (PE) showing *K. pneumoniae* bacterial biofilm on the outer surface. (**b**) SEM micrograph of a polyethylene tracheostomy tube (PE) showing *K. pneumoniae* bacterial biofilm on the inner surface. (**c**) SEM micrograph of a polyvinyl chloride tracheostomy tube (PVC) showing *K. pneumoniae* bacterial biofilm on the outer surface. (**d**) SEM micrograph of a polyvinyl chloride tracheostomy tube (PVC) showing *K. pneumoniae* bacterial biofilm on the inner surface.

SEM revealed the presence of *K. pneumoniae* bacteria adhering to the surfaces of the polyethylene and polyvinyl chloride tracheostomy tubes (Figure 3a–d). The conducted imaging studies revealed that the inner surfaces of both polyethylene and polyvinyl chloride tracheostomy tubes exhibit greater bacterial biofilm growth than the outer surfaces.

## 3. Discussion

As a life-saving procedure, tracheotomy is considered the “gold standard” for securing the patient’s airways and has been carried out for many years, the origins of the technique dating back to ancient times. In addition, the 19th century saw the introduction of various types of tracheostomy tubes [3]. With time, scientists started focusing their interests on the bacterial biofilms forming on the surface of tracheostomy tubes, and the first reports on this subject appeared as early as 1989 [18]. On the other hand, the first literature reports underlining the ability of *K. pneumoniae* to form biofilms on the surface of tracheostomy tubes come from 1988 [19]. It is worth noting that the construction of tracheostomy tubes has really not changed significantly since they were first used; however, there are constant innovations aimed at modifying the physicochemical properties and the structure, texture or composite makeup, with the objective of reducing the risk of bacterial biofilm formation on their inner surface [6].

Several projects are currently underway investigating the impact of tracheal tube design on the prevention of VAP [6,20]. A promising strategy used in the fight against infections associated with the use of biomaterials is the use of coatings made of precious metals with bacteriostatic or bactericidal properties. Coating endotracheal tubes with silver ions has been shown to effectively inhibit biofilm formation and has resulted in significant improvements to the prognoses of intubated ICU patients [21]. A statistically significant reduction in the frequency of VAP episodes in these patients has been indicated [22]. Similar observations also apply to patients using silicone-coated endotracheal tubes (ETTs) [23]. The use of micropatterned ETTs has also shown promising results [24], and in addition, high hopes are placed on the testing of modern equipment (endoClear) for cleaning ETTs [25].

The results presented and discussed below were collected and evaluated with special attention paid to the characteristics of *K. pneumoniae* isolates coming from adult patients with tracheostomies, hospitalized in two hospital units—otolaryngology and Intensive Therapy—in south-eastern Poland. All strains were isolated from secretions (tracheal aspirates) from 18 tracheostomy tubes constituting 34% out of a total of 53 (all tubes collected). In studies on the microbiology of tracheal secretions carried out by the Brazilian researchers El Cheik et al., among all Gram-negative isolates from adults and children with tracheostomy, *K*. *pneumoniae* strains were isolated from 5% [5]. On the other hand, the American scientists Mishra et al. described the predominant pathogens capable of biofilm formation and causing infections associated with different medical devices, of which *K. pneumoniae* accounted for 20% [26]. The results of drug resistance testing of the studied *K. pneumoniae* demonstrated that 33.3% of isolates were resistant to cefuroxime and 16.7% of isolates were resistant to several other marker ESβL-mechanism oxyimino-β-lactams (ceftazidime, cefotaxime, cefepime and aztreonam). Contrarily, the results obtained by an American team of researchers showed that 97% of *K. pneumoniae* isolates were resistant to cefuroxime, 81.5% resistant to aztreonam, 72% resistant to ceftazidime, 68.5% resistant to cefotaxime and 8.9% resistant to cefepime [14]. Meanwhile the analysis conducted by a Mexican research team, Cruz-Cordova et al., found 88% of *K. pneumoniae* strains resistant to aztreonam, 80% resistant to ceftazidime and 26% resistant to cefepime [16]. In the pool of the *K. pneumoniae* tested, in the group of fluoroquinolones, 22.2% of the isolates were resistant to ofloxacin, which contrasts with the study by Cruz Cordova et al., in which 2% of strains were found to be resistant to this antibiotic [16]. Furthermore, the characterization that was carried out found 22.2% of *K. pneumoniae* isolates to be resistant to ciprofloxacin. Similarly, Chinese researchers showed resistance to ciprofloxacin in 26% of isolated *K. pneumoniae* [27]. Studies published by other teams describe the resistance of *K. pneumoniae* to ciprofloxacin, ranging from 6% to 8% [14,16]. Among the aminoglycosides in this study, no strains of *K. pneumoniae* resistant to amikacin were detected, in contrast to the results of groups of scientists from Mexico, who recorded the relatively high percentage of 70% of *K. pneumoniae* isolates as being resistant to this drug [14,16]. The studies carried out by us did not show the presence of *K. pneumoniae* strains resistant to imipenem and meropenem, while in the studies conducted by Cruz-Cordova et al. slightly different results were obtained: 4% of *K. pneumoniae* isolates were resistant to imipenem and 2% of isolates were resistant to meropenem [16]. Due to the fact that the most important mechanism of *K. pneumoniae* resistance to beta-lactam antibiotics is the production of extended-spectrum β-lactamases (ESβL), the study assessed the prevalence of *bla*_TEM_, *bla*_SHV_ and *bla*_CTX-M_-type genes among the ESβL-positive *K. pneumoniae* strains [28,29]. In all three isolates (11%), the presence of *bla*_SHV_, *bla*_TEM_ and *bla*_CTX-M group 1_ was confirmed. Additionally, one isolate had the gene *bla*_CTX-M group 9_. For comparison, in the research conducted by Cruz-Cordova et al., the presence of the ESβL phenotype was confirmed in 97% of the strains [16]. We have also assessed the ability of the *K. pneumoniae* isolates studied to form biofilms. The strains were analyzed in terms of their biofilm production using the crystal violet absorption method [30]. According to the definition proposed in the protocol prepared by Stepanovic et al., biofilm-forming clinical strains are classified into four categories as strong biofilm producers, moderate biofilm producers, weak biofilm producers and non-biofilm producers [31]. The proposed criteria for classifying biofilm producers are also widely used by researchers working with *K. pneumoniae* strains [32,33,34].

In the present study, *K. pneumoniae* strains forming strong biofilm were not detected, 22% of isolates were characterized by average biofilm formation, 22% of isolates produced small biofilm, and 56% of isolates did not form biofilm. For comparison, the study by Dolores Alcántar-Curiel et al., encompassing a collection of 168 hospital isolates of *K. pneumoniae*, demonstrated significant biofilm formation in 69% of isolates, weak biofilm production in 20.3% of isolates, while 10.1% of isolates did not produce biofilm at all [14]. On the other hand, research carried out on 50 isolates of *K. pneumoniae* by Cruz-Cordova et al. (published in 2014), demonstrated that 64% of strains were strong biofilm producers, 26% were moderate, and 10% of isolates were weak producers [16]. Whereas the results published by Seifi et at. showed that, among 94 *K. pneumoniae* analyzed, 33% of the isolates produced fully established biofilms, 52.1% formed biofilms moderately, 8.5% were categorized as weak biofilm-producing, and 6.4% were classified as non-biofilm producers [15]. Adhesive properties of *Klebsiella* are associated with the presence of fimbriae and non-fimbrial adhesion factors in these bacteria [35]. The variety of structural and morphological properties of the fimbrial projections and the specificity of the proper adhesins which are part of their structure may pose some difficulties in their classification. In this study, the presence of the encoding gene *mrk*A was demonstrated in all (100%) isolates of *K. pneumoniae,* while the gene *fim*A was found in 27.8% of the strains. For comparison, the studies conducted by Alcántar-Curiel et al. revealed the presence of *mrk*A in 100% of isolates, while *fim*A was found in 78% of strains [35]. On the other hand, in the aforementioned study, the encoding genes *ecp*A, *mrk*D and *fim*H were found in 84%, 38.9% and 27.8% of isolates, respectively [35]. In this study, *ecp*A was found in 50%, *mrk*D in 20%, and *fim*H in 22% of the strains.

The PFGE molecular method was used to assess the genetic relationship between the *K. pneumoniae* studied, mainly due to the repeatability of the results and the high level of discrimination [36,37]. The conducted analysis revealed a great genetic diversity across *K. pneumoniae* strains isolated from tracheostomy tubes for the study. This result might be explained by the origin of the strains, i.e., from one center, but from sporadic cases of *K. pneumoniae* colonization/infections, not from epidemic outbreaks. Furthermore, the collected isolates were not related epidemiologically. Nevertheless, the analysis carried out among the tested strains made it possible to demonstrate the presence of two pulsotypes (A and B) characteristic of more than one strain each, which may indicate a common origin. Similar results of large genetic diversity among hospital-sourced *K. pneumoniae* examined using the PFGE method were obtained by Alcántar-Curiel et al., who identified among 168 isolates 59 different PFGE profiles [14]. However, the above observations are difficult to discuss with other Polish and international authors due to the small number of studies covering genetic relatedness and concentrating solely on *K. pneumoniae* strains isolated from tracheal secretions from tracheostomy tubes rather than isolates derived from a variety of other clinical materials [1,5,14,36].

In our own investigations using SEM, significantly greater bacterial colonization with *K. pneumoniae* was found on the internal surfaces of the examined biomaterials used in the production of tracheostomy tubes. Similar observations had already been made in 1989 by Inglis et al. [18]. It is worth remembering that all commonly used and available biopolymers affect the tissues of the human body. Without a doubt, the type of material the tracheostomy tube is made of has a significant influence on the adherence of microbial cells and the rate of bacterial biofilm development. Since tracheostomy tubes are movable, they rub against the patients’ tissues, therefore the bacterial biofilm can be mechanically torn off. An examination of the structure of the materials used for the production of tracheotomy tubes carried out by Björling et al., based on scanning electron microscopy, established that tracheostomy prolonged up to 30 days can cause morphological changes on the surface of most tracheostomy tubes made of various biomaterials due to the degradation of polymer chains (with degradation of the polymer chains as a result) [38]. Additionally, the research carried out by this team has demonstrated that silicone tubes are more resistant to degradation than tubes made of polyvinyl chloride or polyurethane [38].

## 4. Materials and Methods

### 4.1. Ethical Approval

The study was approved by the Ethics Committee of the Jagiellonian University Medical College (KBET/1072.6120.153.2019).

### 4.2. Bacterial Strains, Identification and Growth Conditions

Tracheal secretion cultures were collected from 53 tracheostomy tubes (TTs) used to treat adult patients admitted to the Department of Otolaryngology and Oncological Surgery of the Head and Neck Surgery (ORL) and the Department of Anesthesiology and Intensive Therapy (OAIT), 5th Military Hospital with Polyclinic in Krakow (Poland), during a 30-month period, from July 2017 to December 2019. The tested materials were inoculated onto BD^TM^ Columbia Agar with 5% Sheep Blood (BBL, Becton Dickinson, Sparks, MD, USA) and BBL™ MacConkey II Agar (Becton Dickinson, Sparks, MD, USA). Of the total number of samples, 18 grew *K. pneumoniae*, which was identified by the analysis of biochemical profiles of the isolates using the miniAPI System with API 20E test (bioMérieux, Marcy l’Etoile, France), according to the manufacturer’s instructions. All isolates were stored in the Mikrobank™ Preservation System (BIOMAXIMA, Lublin, Poland) at −80 °C for further investigation.

### 4.3. Antibiotic Susceptibility Testing

Antibiotic susceptibility testing was performed by the E-test method according to the European Committee on Antimicrobial Susceptibility Testing (EUCAST) guidelines [39]. The antibiotics (The Liofilchem^®^ MIC Test Strips, Roseto degli Abruzzi, Italy) evaluated included: amikacin (0.016–256 µg/mL); gentamicin (0.016–256 µg/mL); netilmicin (0.016–256 µg/mL); tobramycin (0.016–256 µg/mL); ciprofloxacin (0.002–32 µg/mL); levofloxacin (0.002–32 µg/mL); moxifloxacin (0.002–32 µg/mL); ofloxacin (0.002–32 µg/mL); imipenem (0.002–32 µg/mL); meropenem (0.002–32 µg/mL); ertapenem (0.002–32 µg/mL); doripenem (0.002–32 µg/mL); colistin (0.016–256 µg/mL); trimethoprim-sulfamethoxazole (0.002–32 µg/mL); piperacillin (0.016–256 µg/mL); ceftriaxone (0.016–256 µg/mL); ceftazidime (0.016–256 µg/mL); cefotaxime (0.016–256 µg/mL); cefepime (0.016–256 µg/mL); aztreonam (0.016–256 µg/mL) and cefuroxime (0.016–256 µg/mL). Production of extended spectrum beta-lactamases (ESβLs), including ceftazidime and cefotaxime, was screened with clavulanate by the double disk synergism method according to the CLSI criteria [40]. *K. pneumoniae* ATCC^®^700603™ and *Escherichia coli* ATCC^®^25922™ were used as positive and negative controls, respectively.

### 4.4. Biofilm Production Assay

Biofilm formation in the studied *K. pneumoniae* was evaluated according to the method published by Christensen et al., with a few modifications [24]. Fresh colonies of *K. pneumoniae* isolates were used to inoculate 3 mL BACTO™ Tryptic Soy broth (TSB, Becton Dickinson, Franklin Lakes, NJ, USA). The cultures were incubated aerobically for 24 h at 37 °C. Following incubation, the number of cells in each culture was quantified and adjusted to 0.5 McFarland (1.5 × 108 CFU/mL), then the cultures were diluted 1:100 with fresh medium. A sterile individual plate with 12 flat-bottom polystyrene wells (Corning^®^ Costar^®^, Sigma-Aldrich, Darmstadt, Germany) was filled with 3 mL of the diluted culture. *K. pneumoniae* ATCC^®^700603™ was used as a positive control strain in the biofilm assays and was also processed in a similar manner. Sterile TSB was incorporated as a negative control. The inoculated microtiter plates were incubated on a shaking platform at 37 °C for 48 h. Subsequently, the wells were gently washed three times with Dulbecco’s Phosphate Buffered Saline (DPBS, Lonza, pH = 7.4) to remove free-floating bacteria. Biofilm mass was fixed with methanol and stained with 1% crystal violet (CV, Sigma-Aldrich) for 20 min. The plates were washed exhaustively with distilled water and set aside to dry. Optical density (OD) measurement was performed using a microplate reader Tecan Infinite^®^ 200 PRO (Tecan Group Ltd., Männedorf, Switzerland) at a wavelength of 595 nm supported by the i-control 2.0.10.0. software. The experiment was performed in triplicate. The interpretation for biofilm production was made according to the criteria of Stepanovic et al. [31].

### 4.5. Multiplex PCRs for β-Lactamase Encoding Genes (bla_SHV_, bla_TEM_ and bla_CTX-M-type_)

Genes encoding SHV, TEM extended spectrum β-lactamases (ESβLs) were detected via multiplex polymerase chain reactions (PCRs) [28]. *bla*_CTX-M_-type gene families (*bla*_CTX-M_ group 1, *bla*_CTX-M_ group 9) were detected according to Xu et al. [29].

### 4.6. PCR Detection of Fimbrial Genes

The presence of type 1 and 3 pili and ECP (*ecp*A, *fim*A, *fim*H, *mrk*A and *mrk*D)—major structural fimbrial genes—was determined by DNA amplification using specific primers, as described by other research teams [35]. *K. pneumoniae* ATCC^®^700603™ was employed as a positive control for type 1 and type 3 pili production.

### 4.7. Genotyping by Pulsed-Field Gel Electrophoresis (PFGE)

To determine the genetic relatedness of the studied *K. pneumoniae*, PFGE analysis was performed as previously described [14,36]. Chromosomal DNA of isolates was prepared as described elsewhere [14,26]. DNA fragments were prepared by *Xba*I (Thermo Fisher Scientific, Waltham, MA, USA) restriction analysis and separated by PFGE using a CHEF DR^®^II System (Bio-Rad^®^, Hercules, CA, USA). PFGE profiles were compared according to their percentage of similarity estimated by the Dice coefficient and clustered by UPGMA (unweighted pair-group method with arithmetic averages) using the GelCompar II software, version 6.5 (Applied Maths, Sint-Martens-Latem, Belgium). Restriction fragment analysis was used to define clonal related or unrelated isolates according to the Tenover criteria [37]. *K. pneumoniae* ATCC^®^700603™ was used as a reference strain.

### 4.8. Scanning Electron Microscopy (SEM)

Two types of sterile tracheostomy tubes were used in this study as substrate biomaterials for scanning electron microscopy (SEM) observation: polyethylene (PE, Demed, Mikołów, Poland) and polyvinyl chloride (PVC, SUMI, Sulejówek, Poland). Adult tracheostomy tubes with an internal diameter of 9.5 mm were examined. For negative controls, samples of each tracheostomy tube were placed in TSB without bacteria. The experimental arm consisted of samples of each tracheostomy tube in a separate solution of *K. pneumoniae* alone. For SEM, we followed a protocol established by Jarrett et al. [41] to grow bacteria to a concentration of 106 colony forming units (CFU) per ml. A standardized laboratory strain of *K. pneumoniae* was inoculated in tryptic soy broth (TSB), a nonselective, enriched, liquid growth medium. The bacterial strain was grown to a logarithmic phase over 24 h at 37 °C. Next, the bacteria were harvested by centrifugation at 2000 rpm at 25 °C for 15 min. Three cycles of centrifugation were performed to ensure thorough bacterial isolation. Bacterial isolates were redispersed in 20 mL of TSB in sterile Falcon™ Round-Bottom Polystyrene Tubes and serially diluted to a concentration of 106 CFU/mL based on McFarland turbidity. For negative controls, samples of each tracheostomy tube were placed in TSB without bacteria. The experimental arm consisted of samples of each tracheostomy tube in a separate solution of *K. pneumoniae* alone. All culture tubes were maintained at 37 °C for 48 h to promote biofilm formation. The samples were removed and rinsed ten times with 35 mL of sterile water to remove free bacteria and debris that were not attached to the samples. In preparation for scanning electron microscopy (SEM), each TT fragment was fixed in a 2.5% glutaraldehyde solution (Sigma-Aldrich) in 0.1 M DPBS for 24 h, rinsed with DPBS 2 × 10 min at room temperature and dehydrated in a graded series of ethanol concentrations (50%, 75%, 80%, 96%) (Sigma-Aldrich, Steinheim, Germany) for 10 min in each solution and twice for 30 min in 99.8% ethanol at room temperature. Finally, the samples were placed in a transitional liquid (100% acetone) and transferred to a Critical Point Dryer (CPD E 3000/E3100, Quorum Technologies, Lewes, UK). Ready biomaterials were mounted on aluminum tables, sputter coated with gold particles and examined with a Hitachi S-4700 FE-SEM.

### 4.9. Statistical Analysis

Boxplots were created with the use of STATISTICA (version 13.3, Santa Clara, CA, USA) in order to visualize the mean value of absorbance (od 595 nm) by the tested strains.

## 5. Conclusions

Proven susceptibility of TT biomaterials to colonization by *K. pneumoniae* means that the attention of research groups should be focused on achieving a better understanding of the bacterial pathogens that form biofilms on the surfaces of TTs. In addition, research efforts should focus on the development of new biomaterials or on the modification of existing materials in order to prevent bacterial adhesion to their surfaces.

## Figures and Tables

**Table 1:** Characteristics of patients with tracheostomy, 5th Military Clinical Hospital with Polyclinic, Independent Public. Health Care Facility in Krakow—5WSzKzP SPZOZ, with isolated strain of *K. pneumoniae*.

No.	Patient ID/Isolate No.	Age (Years)/Sex	Hospital Ward(s)	Lenght of Stay in the Ward	Final Diagnosis. ICD9 Treatments/Procedures/Consultation	Antibiotic Treatment
1.	5	98/M	ORL	31.07–03.08.2017	Dyspnoea. A tumor in the larynx. Endoscopic laryngeal biopsy. Emergency tracheostomy. Laryngeal microsurgery using the Kleinsasser instrument set.	Amoxicillin + Clavulanic acid
2.	9	68/M	ORL	11.12–15.12.2018	Laryngeal cancer (Carcinoma planoepitheliale GII). Dyspnoea. Permanent tracheostomy.	Amoxicillin + Clavulanic acid
3.	12	77/M	ORL	20.11–22.12.2018	Laryngeal cancer (Squamous cell carcinoma GIII, Carcinoma planoepitheliale laryngis GIII). Extended excision of regional lymph nodes. Excision of the larynx and pharynx. Esophageal repair procedures.	Amoxicillin + Clavulanic acid, Ciprofloxacin
4.	15	93/F	OAIT	18.10–29.11.2018	Gastrointestinal obstruction. Exploratory laparotomy. Sigmoid tumor excision (adenocarcinoma of the colon). Peroration of the ascending colon in the course of surgery. Relaparotomy. Necrosis of the caecum due to ischemia. Diffuse peritonitis. Acute circulatory and respiratory failure. Multiple organ failure. Dementia syndrome. Chronic respiratory failure. Temporary tracheostomy. Sepsis. Death.	Ampicillin + Sulbactam, Ciprofloxacin, Fluconzole, Gentamicin, Meropenem
5.	18	90/F	OAIT	15.11.2018–03.01.2019	Septic shock. Acute respiratory failure. State after aspiration of gastric contents. Aspiration pneumonia. Status after a stroke of the right hemisphere of the brain. Left-sided hemiparesis. Epilepsy of vascular aetiology. Type 2 diabetes. Goiter of the thyroid gland with displacement of the trachea. Ischemic heart disease. Persistent atrial fibrillation. Coaulopa thy resulting from acenocoumarol overdose. Hypertension. Condition after left femoral neck fracture. State after amputation of the lower right limb. Temporary tracheostomy. Death.	Ampicillin + Sulbactam, Clindamycin, Piperacillin + Tazobactam, Vancomycin
6.	21	75/M	ORL	21.01–08.02.2019	Squamous cell carcinoma of the larynx GII. Status after right-sided pneumectomy due to squamous cell carcinoma of the lung followed by radiotherapy due to cancer infiltration in the bronchial stump. Hypertension. Complete removal of the larynx with simultaneous reconstruction of the cervical esophagus. Tracheostomy.	Amoxicillin + Clavulanic acid, Metronidazole
7.	22	61/M	ORL	23.01–25.01.2019	Carcinoma planoepitheliale laryngis GII.	
8.	23	65/M	ORL	17.01–20.01.2019	Suspicion of recurrence of laryngeal cancer. Status after radiotherapy for laryngeal cancer. Permanent tracheostomy.	Amoxicillin + Clavulanic acid
9.	28	69/M	ORL	01.03–12.03.2019	Carcinoma planoepitheliale laryngis GI. Condition after duodenal ulcer bleeding. Acute haemorrhagic anemia. Hyperten sion. Type 2 diabetes. Condition after myocardial infarction. Total laryngectomy. Removal of the neck lymph nodes on the right side. Esophageal surgery.	Amoxicillin + Clavulanic acid
10.	29	89/M	ORL	06.03–27.03.2019	Recurrence of laryngeal squamous cell carcinoma GII. Condition after radiotherapy and partial surgery. Total laryngectomy. Esophageal surgery.	Amoxicillin + Clavulanic acid
11.	30	74/M	OAIT	15.03–07.04.2019	Acute respiratory failure. Hypercapnia. Respiratory acidosis. Persistent atrial fibrillation. Hypertension. Condition after cholecystectomy. Condition after myocardial infarction. Pulmonary hypertension. Tracheostomy. Puncture of the pleural cavities. Blood transfusion. Respirator therapy.	Colistin, Imipenem, Vancomycin
2.	38	88/F	OAIT	14.05–19.06.2019	Stroke. Chronic respiratory failure. Chronic heart failure. Persistent atrial fibrillation. Condition after ICD implantation. Status after PCI LAD. Hypertension. Pulmonary hypertension. Insulin-dependent type 2 diabetes mellitus. Tracheostomy. Craniotomy for a subarachnoid hematoma.	Ciprofloxacin, Meropenem
13.	42	84/F	OAIT	03.10–28.11.2018	Chronic respiratory failure. Tracheostomy. Acute postoperative respiratory failure. Condition after fronto-temporo-parital craniotomy due to acute subdural hematoma over the right hemisphere. Status after iatrogenic gastrointestinal perforation. Hypertension. Digestive tract cancer (area of the ma jor duodenal papilla). Cancer cachexia. Hypoalbuminemia. History of gastric polyp. Dysphagia. Type 2 diabetes mellitus.	Amoxicillin + Clavulanic acid, Gentamicin, Piperacillin + Tazobactam
14.	43	65/F	ORL	27.11–17.12.2018	Cancer of the middle throat and left side of the tongue (recurrent). State after RTH-CHTH in 2012. Partial pharygetomy. Exploring the neck area through the incision. One-sided radical neck dissection. Throat repair operations other.	Amoxicillin + Clavulanic acid, Ceftazidime, Cefuroxime, Metronidazole
15.	45	95/F	OAIT	23.08–20.09.2019	Oedema pulmonalis, COPD, brain infarction, insulin-dependent type 2 diabetes mellitus. Hypertension. Ischemic heart disease. Alcohol dependence syndrome. Liver damage. Tra cheostomy. Respirator therapy.	Ceftriaxone, Cefuroxime, Levofloxacin
16.	46	70/M	ORL	12.09–14.09.2019	Acute respiratory failure in the course of laryngeal cancer. End-stage renal failure during renal replacement therapy. Hypertension. Ischemic heart disease. Status after STEMI. Secondary hypoparathyroidism. Tracheostomy.	Ampicillin
17.	47	65/M	ORL	26.11–17.12.2019	Recurrence of laryngeal squamous cell carcinoma GII. Condition after partial laryngectomy. Total laryngectomy. Purulent fistula starting 4 days after surgery (*K. pneumoniae* in swab).	Clindamycin, Ceftriaxone, Ciprofloxacin
18.	48	61/M	ORL	27.10–20.11.2019	Laryngeal squamous cell carcinoma GII. Excision of the larynx and pharynx. Bilateral lymph node removal. Esophageal surgery.	Amoxicillin + Clavulanic acid, Ciprofloxacin

CHTH, Chemotherapy; ICD, implantable cardioverter defibrillator; COPD, Chronic obstructive pulmonary disease; LAD, left anterior descending; OAIT, Department of Anaesthesiology and Intensive Therapy; ORL, Department of Otolaryngology and Oncological Surgery of the Head and Neck Surgery; PCI, percutaneous coronary intervention; RTH, radiotherapy; STEMI, ST-segment elevation myocardial infarction.

**Table 2:** Patterns of antibiotic resistance of clinical *K. pneumoniae* strains isolated from tracheal secretions from patients who had undergone a tracheostomy.

Microorganism *Klebsiella pneumoniae*	n (%) 18 (100)	Resistance (%)
**Penicillins**		
Piperacillin	4	22.2
**Cephalosporins**		
Cefepime	3	16.7
Cefotaxime	3	16.7
Ceftazidime	3	16.7
Ceftriaxone	4	22.2
Cefuroxime	6	33.3
**Monobactams**		
Aztreonam	3	16.7
**Fluoroquinolones**		
Ciprofloxacin	4	22.2
Levofloxacin	4	22.2
Moxifloksaxin	4	22.2
Ofloxacin	4	22.2
**Aminoglicosides**		
Gentamycin	1	5.5
Netylmycin	2	11
Tobramycin	3	16.7
**Miscellaneous agents**		
Trimethoprim-sulfamethoxazole	5	27.8

## Data Availability

Data sharing not applicable.

## References

[1] Cader S.H.A., Shah F.A., Nair S.K.G.R. (2020). Tracheostomy colonisation and microbiological isolates of patients in intensive careunits—A retrospective study. World J. Otorhinolaryngol.-Head Neck Surg..

[2] Weissbrod P.A., Merati A.L. (2012). Is percutaneous dilational tracheotomy equivalent to traditional open surgical tracheotomy with regard to perioperative and postoperative complications?. Laryngoscope.

[3] Szmuk P., Ezri T., Evron S., Roth Y., Katz J. (2008). A brief history of tracheostomy and tracheal intubation, from the Bronze Age to the Space Age. Intensive Care Med..

[4] Percival S.L., Suleman L., Vuotto C., Donelli G. (2015). Healthcare-Associated infections, medical devices and biofilms: Risk, tolerance and control. J. Med. Microbiol..

[5] El Cheikh M.R., Barbosa J.M., Caixêta J.A.S., Avelino M.A.G. (2018). Microbiology of tracheal secretions: What to expect with children and adolescents with tracheostomies. Int. Arch. Otorhinolaryngol..

[6] Rouzé A., Jaillette E., Poissy J., Préau S., Nseir S. (2017). Tracheal tube design and ventilator-associated pneumonia. Respir. Care..

[7] Chastre J., Fagon J. (2002). State of the Art Ventilator-associated Pneumonia. Am. J. Respir. Crit. Care Med..

[8] Lepainteur M., Ogna A., Clair B., Dinh A., Tarragon C., Prigent H., Davido B., Barbot F., Vaugier I., Afif M. (2019). Risk factors for respiratory tract bacterial colonization in adults with neuromuscular or neurological disorders and chronic tracheostomy. Respir. Med..

[9] McAfee J.S., Demarcantonio M., Fine B.R., Beydoun H., Derkay C. (2013). Prevalence of ventilation tubes in children with a tracheostomy tube. Int. J. Pediatr. Otorhinolaryngol..

[10] Chung P.Y. (2016). The emerging problems of infections: Carbapenem resistance and biofilm formation. FEMS Microbiol. Lett..

[11] Bryers J.D. (2008). Medical biofilms. Biotechnol. Bioeng..

[12] Piperaki E.T., Syrogiannopoulos G.A., Tzouvelekis L.S., Daikos G.L. (2017). *Klebsiella pneumoniae:* Virulence, Biofilm and Antimicrobial Resistance. Pediatr. Infect. Dis. J..

[13] Solomon D.H., Wobb J., Buttaro B.A., Truant A., Soliman A.M.S. (2009). Characterization of bacterial biofilms on tracheostomy tubes. Laryngoscope.

[14] Alcántar-Curiel M.D., Ledezma-Escalante C.A., Jarillo-Quijada M.D., Gayosso-Vázquez C., Morfín-Otero R., Rodríguez-Noriega E., Cedillo-Ramirez M.L., Santos-Preciado J.I., GirÓn J.A. (2018). Association of Antibiotic Resistance, Cell Adherence, and Biofilm Production with the Endemicity of Nosocomial *Klebsiella pneumoniae*. BioMed Res. Int..

[15] Seifi K., Kazemian H., Heidari H., Rezagholizadeh F., Saee Shirvani F., Houri H. (2016). Evaluation of biofilm formation among *Klebsiella pneumoniae* isolates and molecular characterization by ERIC-PCR. Jundishapur J. Microbiol..

[16] Cruz-Córdova A., Esteban-Kenel V., Espinosa-Mazariego K., Ochoa S.A., Espinosa M., de la Garza D., Fenández RendÓn E., LÓpez Villegas E.O., Xicothencotl-Cortes J. (2014). Hospital Infantil de México Pathogenic determinants of clinical *Klebsiella pneumoniae* strains. Bol. Med. Hosp. Infant. Mex..

[17] Samia B., Hafida H., Damien B., Nicolas C., Imane M.H., Ibtissem K.T., Merieme L., Wafae D., Christiane F. (2013). Evaluation of biofilm formation of *Klebsiella pneumoniae* isolated from medical devices at the University Hospital of Tlemcen, Algeria. African J. Microbiol. Res..

[18] Inglis T.J.J., Millar M.R., Jones J.G., Robinson D.A. (1989). Tracheal tube biofilm as a source of bacterial colonization of the lung. J. Clin. Microbiol..

[19] LeChevallier M.W., Cawthon C.D., Lee R.G. (1988). Inactivation of biofilm bacteria. Appl. Environ. Microbiol..

[20] Poelaert J., Pieter Depuydt P., De Wolf A., Van de Velde S., Herck I., Blot S. (2008). Polyurethane cuffed endotracheal tubes to prevent early postoperative pneumonia after cardiac surgery: A pilot study. J. Thorac. Cardiovasc. Surg..

[21] Mahmodiyeh B., Kamali A., Zarinfar N., Mohammadi Joushani M. (2021). The Effect Of Silver-Coated Endotracheal Tube On The Incidence Of Ventilator-Induced Pneumonia In Intubated Patients Admitted To The Intensive Care Unit (ICU). Syst. Rev. Pharm..

[22] Kollef M.H., Afessa B., Anzueto A., Veremakis C., Kerr K.M., Margolis B.D., Craven D.E., Roberts P.R., Arroliga A.C., Hubmayr R.D. (2008). Silver-coated endotracheal tubes and incidence of ventilator-associated pneumonia: The NASCENT randomized trial. JAMA.

[23] Thorarinsdottir H.R., Kander T., Holmberg A., Petronis S., Klarin B. (2020). Biofilm formation on three different endotracheal tubes: A prospective clinical trial. Crit. Care.

[24] Mann E.E., Magin C.M., Mettetal M.R., May R.M., Henry M.M., Deloid H., Prater J., Sullivan L., Thomas J.G., Twite M.D. (2016). Micropatterned Endotracheal Tubes Reduce Secretion-Related Lumen Occlusion. Ann. Biomed. Eng..

[25] Pinciroli R., Mietto C., Piriyapatsom A., Chenelle C.T., Thomas J.G., Pirrone M., Bry L., Wojtkiewicz G.R., Nahrendorf M.P., Kacmarek R.M. (2016). Endotracheal Tubes Cleaned With a Novel Mechanism for Secretion Removal: A Randomized Controlled Clinical Study. Respir. Care..

[26] Mishra S.K., Basukala P., Basukala O., Parajuli K., Pokhrel B.M., Rijel B.P. (2014). Detection of Biofilm Production and Antibiotic Resistance Pattern in Clinical Isolates from Indwelling Medical Devices. Curr. Microbiol..

[27] Zheng J., Lin Z.W., Chen C., Chen Z., Lin F.J., Wu Y., Yang S.-Y., Sun X., Yao W.-M., Li D.-Y. (2018). Biofilm formation in *K**lebsiella pneumoniae* bacteremia strains was found to be associated with CC23 and the presence of *wca*G. Front. Cell Infect. Microbiol..

[28] Latifpour M., Gholipour A., Damavandi M.S. (2016). Prevalence of extended-spectrum beta-lactamase-producing *Klebsiella pneumoniae* isolates in nosocomial and community-acquired urinary tract infections. Jundishapur J. Microbiol..

[29] Xu L., Ensor V., Gossain S., Nye K., Hawkey P. (2005). Rapid and simple detection of bla CTX-M genes by multiplex PCR assay. J. Med. Microbiol..

[30] Christensen G.D., Simpson W.A., Younger J.J., Baddour L.M., Barrett F.F., Melton D.M., Beachey E.H. (1985). Adherence of coagulase-negative staphylococci to plastic tissue culture plates: A quantitative model for the adherence of staphylococci to medical devices. J. Clin. Microbiol..

[31] Stepanović S., Vuković D., Hola V., Di Bonaventura G., Djukić S., Ćirković I., Ruzicka F. (2007). Quantification of biofilm in microtiter plates: Overview of testing conditions and practical recommendations for assessment of biofilm production by staphylo-484 cocci. Apmis.

[32] Hassan A., Usman J., Kaleem F., Omair M., Khalid A., Iqbal M. (2011). Evaluation of different detection methods of biofilm formation in the clinical isolates. Braz. J. Infect. Dis..

[33] Kuinkel S., Acharya J., Dhungel B., Adhikari S., Adhikari N., Thapa Shrestha U., Megha Raj Banjara M., Raj Rijal K., Ghimire P. (2021). Biofilm Formation and Phenotypic Detection of ESBL, MBL, KPC and AmpC Enzymes and Their Coexistence in *Klebsiella* spp. Isolated at the National Reference Laboratory, Kathmandu, Nepal. Microbiol. Res..

[34] Silva B.S.N., Alves G.V.P., de Andrade Marques L., Silva S.F.S., de Oliveira Faria G., de Araújo B.L., dos Santos Pedroso R., Penatti A.P.M., de Paula Menezes R., von Dolinger de Brito R. (2021). ‘Duantification of biofilm produced by clinical, environment and hands’ isolates Klebsiella species using colorimetric and classical methods. J. Microbiol. Methods.

[35] Alcántar- Curiel M.D., Blackburn D., Saldaña Z., Gayosso-Vázquez C., Iovine N., De la Cruz M.A., GirÓn J.A. (2013). Multifunctional analysis of *Klebsiella pneumoniae* fimbrial types in adherence and biofilm formation. Virulence.

[36] Han H., Zhou H., Li H., Gao Y., Lu Z., Hu K. (2013). Optimization of Pulse-Field Gel Electrophoresis for Subtyping of *Klebsiella pneumoniae*. Int. J. Enviromental Res. Public Health.

[37] Tenover F.C., Arbeit R.D., Goering R.V., Mickelsen P.A., Murray B.E., Persing D.H., Swaminathan B. (1995). Interpreting chromosomal DNA restriction patterns produced by pulsed-field gel electrophoresis: Criteria for bacterial strain typing. J. Clin. Microbiol..

[38] Björling G., Axelsson S., Johansson U.B., Lysdahl M., Markström A., Schedin U., Aune R.E., Frostell C., Karlsson S. (2007). Clinical use and material wear of polymeric tracheostomy tubes. Laryngoscope.

[39] EUCAST European Committee on Antimicrobial Susceptibility Testing Breakpoint Tables for Interpretation of MICs and Zone Diameters. Version 9.0, Valid from 2019-01-01. http//www.eucast.org.

[40] CLSI (2018). Performance Standards for Antimicrobial Susceptibility Testing.

[41] Jarrett W.A., Ribes J., Manaligod J.M. (2002). Biofilm formation on tracheostomy tubes. Ear Nose Throat J..

